# Comparison of Hydrogen Peroxide Secretion From Living Cells Cultured in Different Formats Using Hydrogel-Based LSPR Substrates

**DOI:** 10.3389/fbioe.2022.869184

**Published:** 2022-04-08

**Authors:** Yang-Jyun Siao, Chien-Chung Peng, Yi-Chung Tung, Yih-Fan Chen

**Affiliations:** ^1^ Institute of Biophotonics, National Yang Ming Chiao Tung University, Taipei, Taiwan; ^2^ Research Center for Applied Sciences, Academia Sinica, Taipei, Taiwan

**Keywords:** localized surface plasmon resonance, agarose gel, hydrogen peroxide, enzyme-catalyzed reaction, 3D cell culture, microfluidics

## Abstract

Reactive oxygen species (ROS), a number of reactive molecules and free radicals derived from molecular oxygen, are generated as by-products during mitochondrial electron transport within cells. Physiologically, cells are capable of metabolizing the ROS exploiting specific mechanisms. However, if excessive ROS accumulate inside the cells, it will cause the cells apoptosis or necrosis. Hydrogen peroxide (H_2_O_2_) is one of the essential ROS often participating in chemical reactions in organisms and regulating homeostasis in the body. Therefore, rapid and sensitive detection of H_2_O_2_ is a significant task in cell biology research. Furthermore, it has been found that cells cultured in different formats can result in different cellular responses and biological activities. In order to investigate the H_2_O_2_ secretion from the cells cultured in different formats, a hydrogel-based substrate is exploited to separate relatively large molecular (e.g., proteins) for direct measurement of H_2_O_2_ secreted from living cells in complete cell culture medium containing serum. The substrate takes advantage of the localized surface plasmon resonance (LSPR) method based on enzyme immunoprecipitation. In addition, the H_2_O_2_ secreted from the cells cultured in different dimensions (suspension of single cells and three-dimensional cell spheroids) treated with identical drugs is measured and compared. The spheroid samples can be prepared with ample amount using a designed microfluidic device with precise control of size. The results show that the H_2_O_2_ secretion from the cells are great affected by their culture formats.

## Introduction

Reactive oxygen species (ROS) are molecules with highly reactive free oxygen radicals, and their important effects on biological activities have been investigated and identified (Gough and Cotter, 2011; Milkovic et al., 2019). Among various ROS, hydrogen peroxide (H_2_O_2_), a stable non-radical oxidant, has been intensively studied. H_2_O_2_ has been found to increase oxidative stress and damage cells for its excessive production during cell metabolism of aerobic organisms (Gough and Cotter, 2011). Recently, it is discovered that H_2_O_2_ can also act as a beneficial signaling molecules in certain cells (Nindl et al., 2004). As a result, it is essential to monitor H_2_O_2_ secreted from the cells in a time-lapse manner for better investigation the roles of H_2_O_2_ in cellular activities under various stimulations.

*In vitro* cell culture models have been broadly exploited to investigate underlying mechanisms of the H_2_O_2_ secretion due to their well-controlled culture conditions and microenvironments (Hirsch et al., 2014; Queiroz et al., 2021). Recent studies have shown that the cell culture formats can greatly affect the cellular responses under various stimulations. For example, drug treatments on cancer cells cultured in monolayer and three-dimensional (3D) spheroid formats have distinct efficacy and cause different cellular responses (Tung et al., 2011; Patra et al., 2016). In addition, cells cultured in different formats also show different cytokine secretion patterns in temporal domain (Sarkar et al., 2020). Therefore, it is desired to compare the H_2_O_2_ secretion profiles between different culture formats to better investigate the underlying molecular mechanisms related to ROS in a more physiological-like microenvironment.

In order to compare temporal H_2_O_2_ secretion patterns from the cells in different formats, we develop an integrated approach taking advantage of microfluidic cell culture devices, hydrogel-based substrates and LSPR detection in this paper. Microfluidic devices have been broadly exploited for various cell culture applications due to their advantageous properties including: laminar flow, small sample and reagent volume requirement, and great spatiotemporal controllability (Chen et al., 2011; Peng et al., 2013; Polidoro et al., 2021; Zamprogno et al., 2021). Recently, three-dimensional spheroid culture has obtained increasing attention due to its capability of reconstituting three-dimensional physiological microenvironments *in vitro* (Torisawa et al., 2007; Hsiao et al., 2009; Patra et al., 2013; Patra et al., 2014; Patra et al., 2016; Mulas et al., 2020). Consequently, various microfluidic spheroid cultured devices have been constructed for cell studies. In this paper, our previously developed microfluidic device capable of reliably forming and culturing a number of spheroids with uniform sizes is utilized for the spheroid sample preparation (Patra et al., 2013; Patra et al., 2014; Patra et al., 2016). The spheroids can then be harvested from the devices for the treatments and H_2_O_2_ analysis.

For consistent time-lapse H_2_O_2_ measurement on the cell culture medium, a hydrogel substrate with LSPR-based detection scheme is constructed based on our previous research (Chen et al., 2021). Comparing to other commonly used detection schemes such as electrochemical measurement (Liu et al., 2017; Sun et al., 2017; Xin et al., 2017; Zhang et al., 2017; Zhao et al., 2019), field effect transistor (FET) (Xie et al., 2016), and imaging of fluorescent dyes (Feng et al., 2017; Velusamy et al., 2019), the LSPR-based detection can be performed with great consistency in a time-effective manner using a simple optical detection setup. In addition, the hydrogel-based substrate can help to alleviate the interference from other soluble factors contained in the complete cell culture medium to improve the detection sensitivity. Furthermore, incorporating the hydrogel with the enzymatic activity of horseradish peroxidase (HRP), we have demonstrated that the substrate can detect H_2_O_2_ with great specificity. As a result, the temporal profiled of H_2_O_2_ secreted from the cells cultured in different formats can be characterized in a straightforward manner without tedious medium filtration or purification processes (Chen et al., 2021).

In the experiments, human hepatocellular carcinoma cell line is cultured, and H_2_O_2_ secretin of the cells in two formats: single cell and three-dimensional (3D) spheroid are characterized. It has been identified that H_2_O_2_ can induce hepatocyte apoptosis by mechanisms different from other superoxide anions (Conde de la Rosa et al., 2006; Ma et al., 2015). In contrast, it also suggested that moderate transient increase in H_2_O_2_ production can mediate stress adaption and improve cell survival (Miller et al., 2019). Therefore, it is desired to investigate the transient response of the hepatocytes in H_2_O_2_ production under stimulation. For (Miller et al., 2019) comparison, the cells are stimulated using ascorbic acid to promote their H_2_O_2_ secretion, and the time-lapse H_2_O_2_ concentrations in complete culture medium within 6 min after the stimulation are measured every 30 s. The results show that the H_2_O_2_ in complete medium with single cells and cell spheroids cultured in it can be successfully detected, and the culture formats affect the temporal variation of H_2_O_2_ concentrations. The integrated approach provides a straightforward platform to understand the dynamic secretion patterns of H_2_O_2_ from the cells cultured in different formats, and further explore the roles of H_2_O_2_ in carious physiological and pathological microenvironments.

## Materials and Methods

### Hydrogel Substrate Preparation and Optical Setup for H_2_O_2_ Detection

**FIGURE 1:**
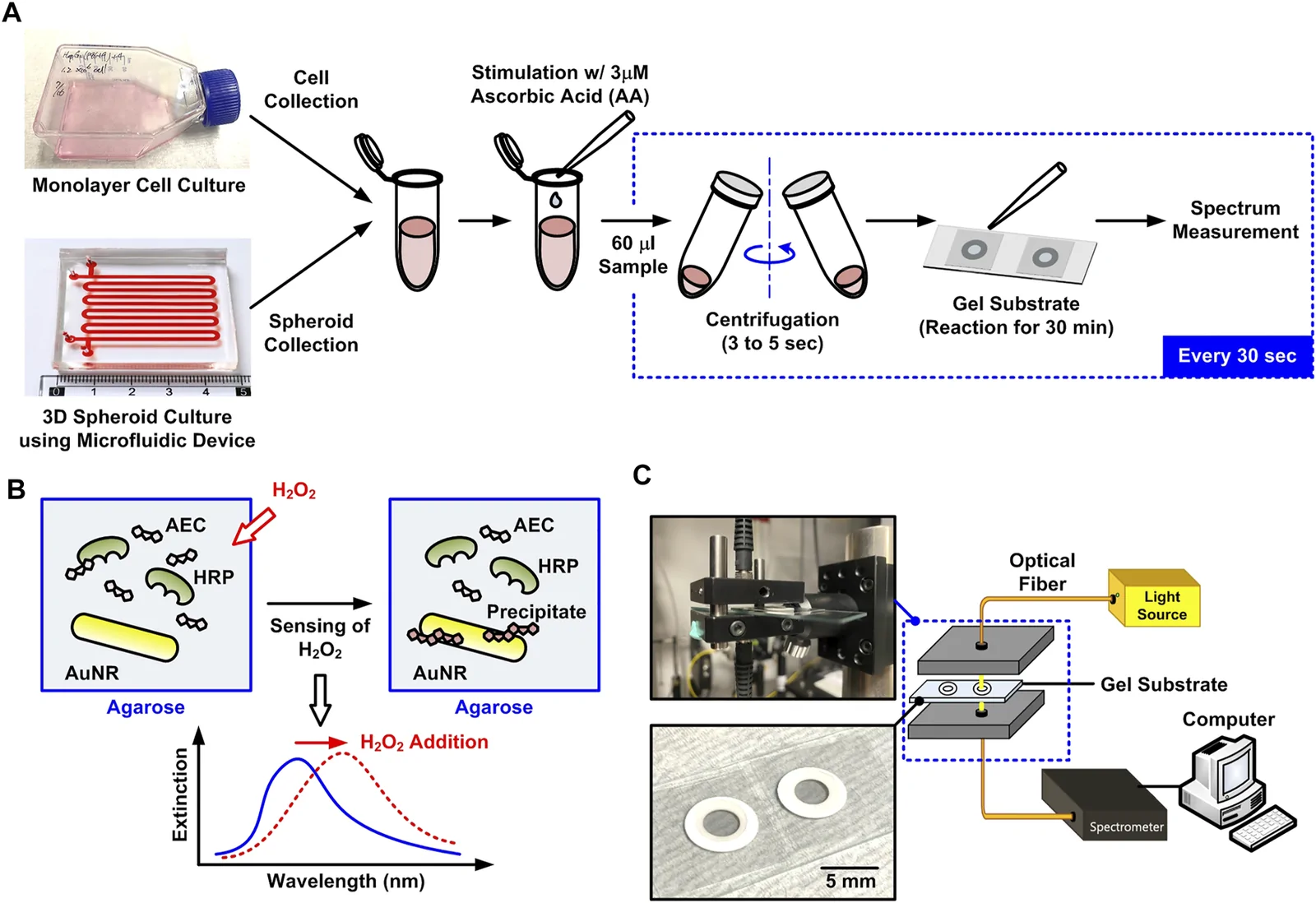
**(A)** Schematic flowchart of the experimental procedures to compare the amount of H_2_O_2_ secreted from the living cells cultured in different formats using the plasmonic gel films with optical spectrum measurement. **(B)** Principle of the H_2_O_2_ detection using the gel film mixed with agarose gel, gold nanorods, AEC and HRP. **(C)** Illustration and photos of the experimental optical spectrum measurement setup for the H_2_O_2_ detection.

In order to perform time-lapse detection of H_2_O_2_ secreted from living cells cultured in different formats, a hydrogel-based substrate capable of generating optical spectrum shift while reacting with H_2_O_2_ due to LSPR and enzyme immunoprecipitation is exploited in the experiments as shown in Figure 1A. The substrate is prepared by mixing gold nano-rods (AuNRs) (NR-40-650, NanoSeedz, Hong Kong), 3-amino-9-ethylcarbazole (AEC) (Sigma-Aldrich), HRP (Sigma-Aldrich), and agarose (Sigma-Aldrich) with final concentrations of 8.48 × 10^10^/ml, 0.125% (v/v), 1.25 μg/ml, and 1.5% (w/v), respectively. In the substrate, the HRP-catalyzed chromogenic reaction forms red precipitates near the AuNRs embedded in the substrate when reacting with H_2_O_2_. The reaction is exploited to generate the spectral shift as shown in Figure 1B. The porous agarose hydrogel mixed in the substrate can stabilize the nanorods and further minimize interferences from other molecules in complex environments like complete cell culture medium. The hydrogel substrate has been tested using the H_2_O_2_ of different concentrations in pure water, DPBS, and complete medium with 10% FBS in our previous research. The results show that the measured LSPR wavelength shifts obtained from the three solutions are identical confirming the hydrogel substrate is capable of alleviating the interference from soluble factors (Chen et al., 2021), and also suggest that the LSPR wavelength shift is insensitive to the refractive index variation caused by the composition and biomolecules in the medium. The entire substrate is fabricated on a microscope slide, and the mixed hydrogel is placed on the slide and confined by layers of ring-shaped self-adhesive reinforcement labels (thickness: ∼290 μm) as shown in Figure 1C. The detail fabrication process of the substrate is described in our previously published paper (Chen et al., 2021).

When H_2_O_2_ in the sample solution reacts with AEC and HRP, the enzymatic reaction produces red water-insoluble precipitates resulting in a shift in the LSPR wave-length of the AuNRs. As a result, comparing the extinction spectra before and after adding a sample solution, the H_2_O_2_ concentration can be quantified by the wavelength shift. In the experiments, an optical fiber-based system is constructed for the spectrum measurement as shown in Figure 1C. In the setup, a broadband tungsten light source (SL1, Stellarnet Inc.) is guided through an optical fiber to illuminate the substrate, and the transmitted light is collected by another optical fiber and guided to a spectral meter (HRS-BD1-025, Mightex, Toronto, ON, Canada) for the spectrum detection. The quantitative data of the detected spectrum is then transmitted to a personal computer to analyze the LSPR wavelength shift using a LabVIEW program. The AuNR used in the experiments has the longitudinal LSPR peak at approximately 650 nm. Therefore, the wavelength shift around 650 nm is used to calculate the wavelength shift. The substrate has been tested to possess great specificity even with addition of other soluble factors [glucose (GLU), glutathione (GSH), dopamine (DA), and uric acid (UA)] and ROS [TBHP, hypochlorite (OCl^−^), superoxide (O_2_^−^), NO, •OH] in complete cell culture medium (Chen et al., 2021).

### Cell Culture

In order to compare the H_2_O_2_ secretion from the single cells and cells in 3D spheroid formats, human hepatocellular carcinoma cells (HepG2, 60025, Bioresource Collection and Research Center, Hsinchu, Taiwan) are exploited in the experiments. The stocks of the cells are cultured using MEM (Gibco 41090-036, Invitrogen Co., Carlsbad, CA)-based culture medium with 10% v/v fetal bovine serum (FBS) (Gibco 10082, Invitrogen Co.), 1% v/v Antibiotic-Antimycotic (Gibco 15240, Invitrogen Co.), 1% v/v sodium pyruvate (Gibco 11360, Invitrogen Co.), and 1% v/v non-essential amino acids (Gibco 11140, Invitrogen Co.) in a humidified cell incubator maintained at 37°C with 5% CO_2_. The cells are passaged every 3 days during the experiments.

The single cells and the cell spheroids used in the experiments are first prepared by culturing cells in conventional monolayer and 3D spheroid formats as shown in Figure 1A. For the monolayer cell culture, cells are cultured in T25 or T75 flasks. The single cells are prepared by dissociating the cells using 0.25% trypsin-EDTA (Gibco 25200, Invitrogen Co.), and the dissociated cells are centrifuged at 1,000 rpm for 5 min. The cells are then resuspended into 1 ml culture medium, and 20 μl of the cell suspension is pipetted into a hemocytometer to evaluate the cell density. The remaining cell suspension containing single cells is adjusted to the density of 1.6 × 10^7^ cells/ml for the H_2_O_2_ measurement experiments.

**FIGURE 2:**
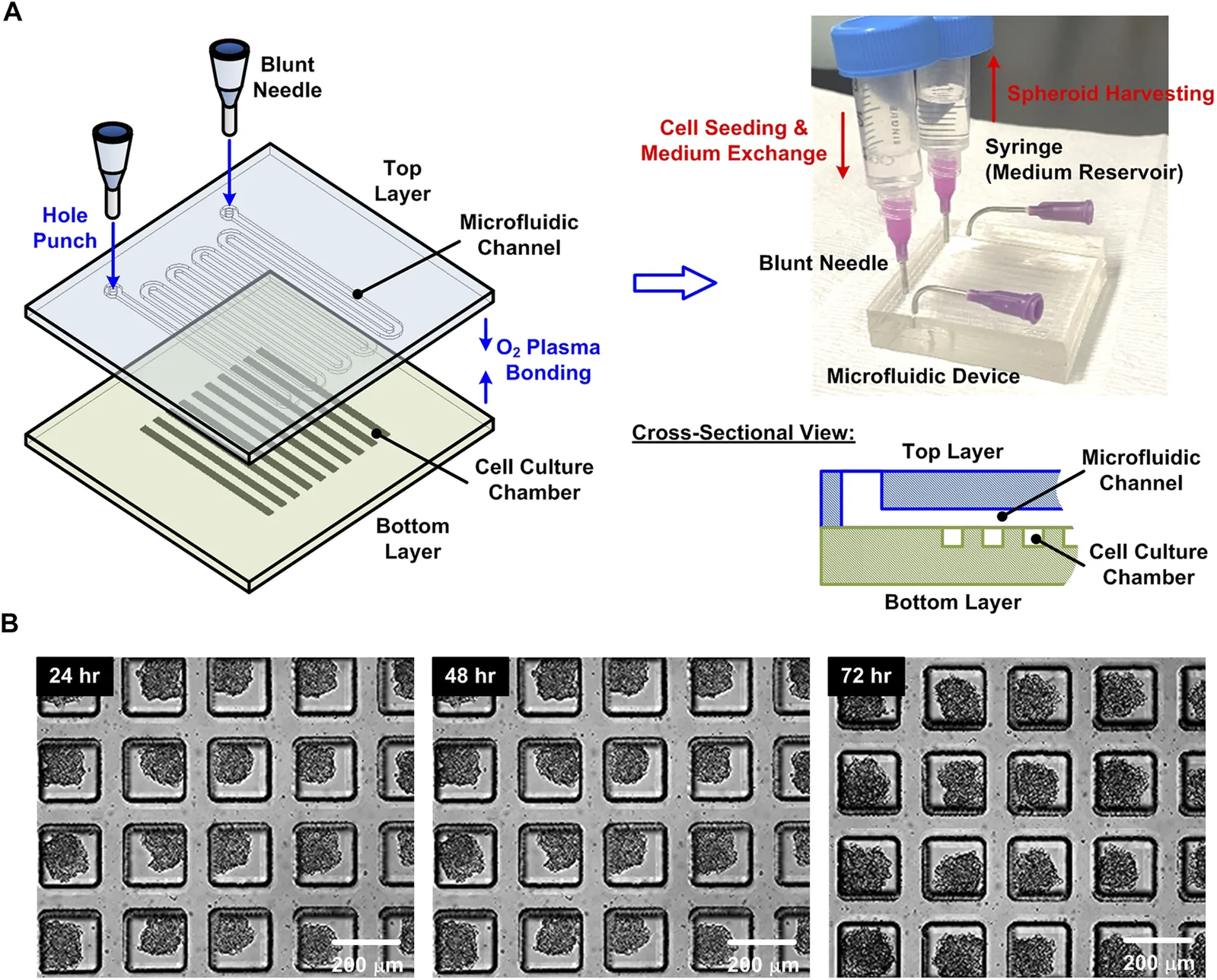
**(A)** Fabrication process, schematic illustration, and experimental photo of the microfluidic device for the 3D cell spheroid formation, culture, and harvesting. **(B)** Experimental photos of the HepG2 cell spheroids formed and cultured within the device for 24, 48, and 72 h, respectively.

For the 3D cell spheroid experiments, the HepG2 spheroids are formed and cultured in a microfluidic device previously designed in our labs as shown in Figure 2A (Patra et al., 2013). In brief, the device is made of an elastomeric material, polydimethylsiloxane (PDMS), due to its optical transparency, gas permeability, and cell compatibility (Xia and Whitesides, 1998; Whitesides et al., 2001). The device is designed with two PDMS layers: a bottom layer with 4,000 cell culture chambers (length × width × depth: 200 μm × 200 μm × 500 μm), and a top layer with a serpentine channel (depth: 200 μm) covering the cell culture chambers. The two layers are bonded using oxygen plasma surface treatment, and the channel walls are coated with 1% w/v Synperonic F-108 (07579, Fluka, Sigma- Aldrich, Co., St Louis, MO) to make them resistant to cell adhesion. To form the spheroids, 200 μl cell suspension with total 8 × 10^6^ cells is introduced into the device, and the growth medium is exchanged every 12 h to maintain the optimized culture condition (Patra et al., 2013). After three day-culture within the device as shown in Figure 2B, the spheroids are harvested from the device by introducing the 20 ml medium with a high flow rate (>1 ml/min). For cell number evaluation, the sample is concentrated to 1.5 ml by centrifugation (700 rpm for 5 min), and the 60 μl well mixed cell suspension with the harvested spheroids is then collected. The cell suspension is diluted using Dulbecco’s phosphate-buffered saline (DPBS) (Gibco 14190, Thermo Fisher Scientific Inc.) to a final volume of 1.5 ml. The saline of the suspension is replaced by cell aggregate dissociation medium (Accumax, 00-4666-56, Thermo Fisher Scientific Inc.) and incubated at 37°C for 15 min to dissociate the spheroids in single cells for cell density evaluation using the hemocytometer. The remaining cell suspension is adjusted to the same cell density used in the single cell experiments (1.6 × 10^7^ cells/ml) by centrifugation (700 rpm for 5 min) and resuspension in the medium for the H_2_O_2_ measurement experiments.

### Cell Treatment and H_2_O_2_ Detection

To observe the difference of H_2_O_2_ secretion from the single cells and cell spheroids, ascorbic acid (AA) (A17759, Alfa Aesar) is used to treat the cells (Chen et al., 2021). In the experiments, 3 μm AA with the same volume of the cell suspension is added into the suspension to yield the final AA concentration of 1.5 μm in the suspension solution for the cell treatment. In order to monitor the secretion of H_2_O_2_ after the treatment in a time-lapse manner, 60 μl of the cell suspension is collected and pipetted onto the hydrogel substrate right after (t = 0 s) and every 30 s for 4 min after the AA addition. The sample is reacted with the substrate for 30 min before the measurement. Each measurement is performed at five different locations on the hydrogel substrate to alleviate the variation resulted from the uneven distribution of the sample or the substrate compositions.

### Fluorescence Staining of the Cells

The viabilities of cells cultured in the flasks, before and after the treatment in different cell formats are checked using a live/dead stain solution containing calcein AM (1 μm) and ethidium homodimer-1 (2 μm) from LIVE/DEAD Viability/Cytotoxicity Kit (L3224, Invitrogen) to ensure the cell compatibility of the experiments and investigate the possible effects from the cell viability on the H_2_O_2_ secretion.

## Results

### Cell Culture and Viability

**FIGURE 3:**
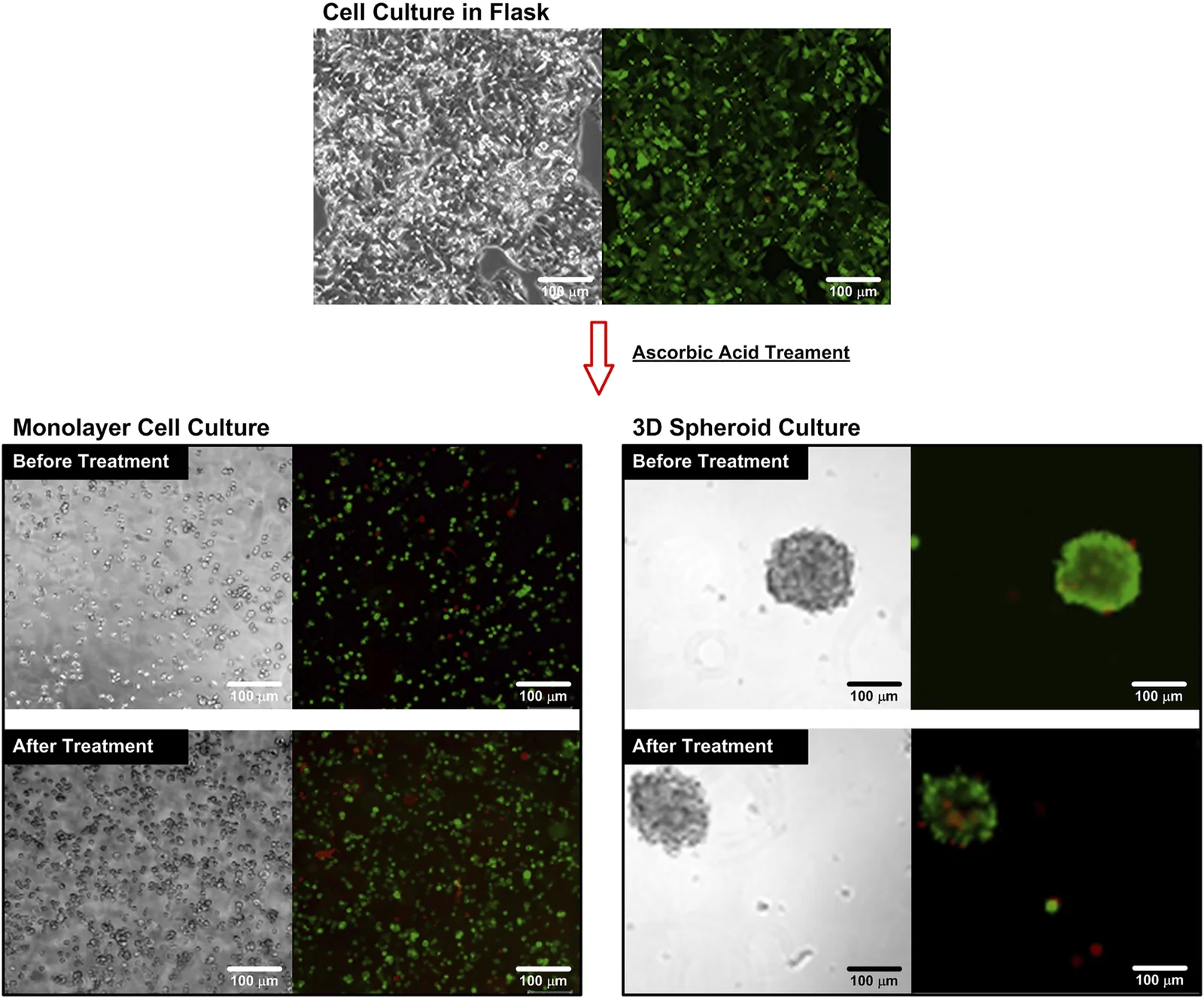
Brightfield and fluorescence microscopy images of the cells cultured in a flask and a microfluidic device before and after the AA treatment. The cells are stained with cell viability kit. The live cells are stained with calcein AM (green), while the dead cells are stained with ethidium homodimer-1 (EthD-1) (red).

In order to ensure that the cells can maintain their live status during the entire experiments, the cell viability assays are performed and the results are shown in Figure 3. The brightfield and fluorescence images of the cells cultured in the flask show that the cells can well attach on the flask surface and most of the cells show green fluorescence from the live cell stain, calcein Am. The cell viability is estimated to be higher than 96% for all the experiments confirming the optimized culture conditions of the culture protocol.

For the single cell experiments, the cultured cells are dissociated into single cells and stained using the cell viability kit before and after the ascorbic acid treatments to observe the effects of the treatments. The fluorescence images (Figure 3) show that most of the cells are alive before and after the treatment. The quantitative average cell viabilities are estimated to be 95.1% and 93.2% with statistical difference (Student’s t-test, *p* < 0.001, *n* = 3) before and after the treatment, respectively. The lower cell viability suggests that the additional of ascorbic acid slightly differ the optimized cell culture conditions, which needs to be carefully examined for the following experiments. For comparison, the cells are also stained using the cell viability kit while they are in the spheroid format for the 3D cell spheroid experiments. The brightfield and fluorescence images show the great integrity of the spheroids, and most of the cells show green fluorescence (calcein-AM) indicating their live status before and after the treatments. The average cell viabilities are estimated to be 92.1% and 91.0% without statistical difference (Student’s t-test, *p* > 0.05, *n* = 3), respectively. The results suggest that the ascorbic acid treatment has minimal effects on the viability of the cells cultured in the spheroid format, which is different from the observation in the single cell experiments.

### H_2_O_2_ Measurement

**FIGURE 4:**
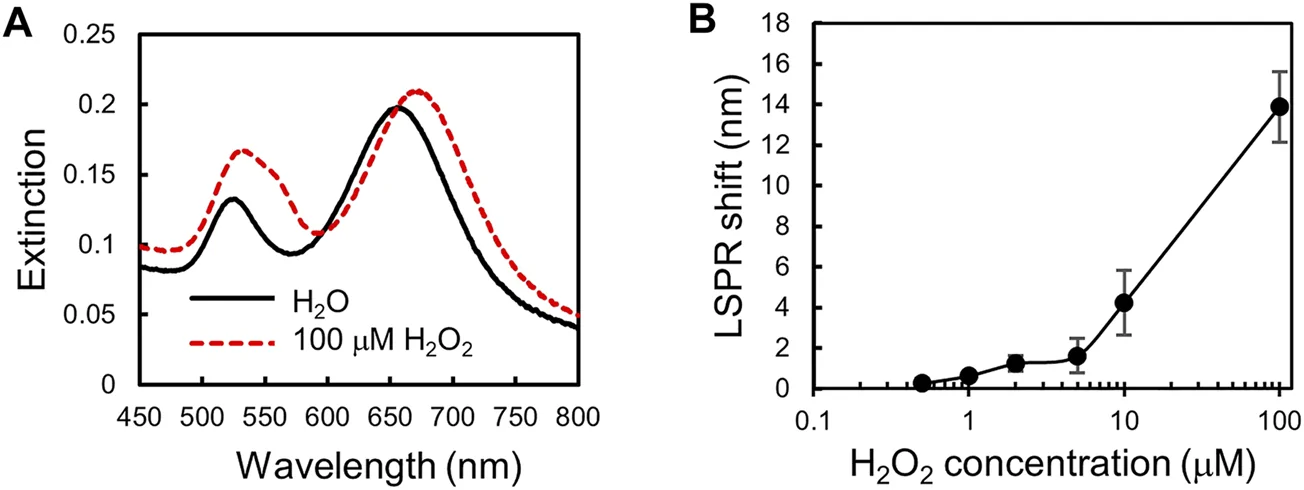
Calibration curve for H_2_O_2_ detection. **(A)** Extinction spectra of the plasmonic gel film be-fore and after incubation with 100 μm H_2_O_2_. **(B)** A calibration curve for H_2_O_2_ detection. A plot of LSPR shift versus H_2_O_2_ concentration. Error bars show standard deviations with *n* = 5.

We measure H_2_O_2_ of various concentrations in MEM medium supplemented with 10%FBS to obtain a calibration curve for H_2_O_2_ detection. Figure 4A shows that the extinction spectra of the plasmonic gel film red-shifted after incubation with 100 μm H_2_O_2_. The LSPR shift of the gel film is different for different concentrations of H_2_O_2_ as shown in Figure 4B. The calibration curve shows that the LSPR shift increases with the concentration of H_2_O_2_ in the range of 0.5–100 μm.

**FIGURE 5:**
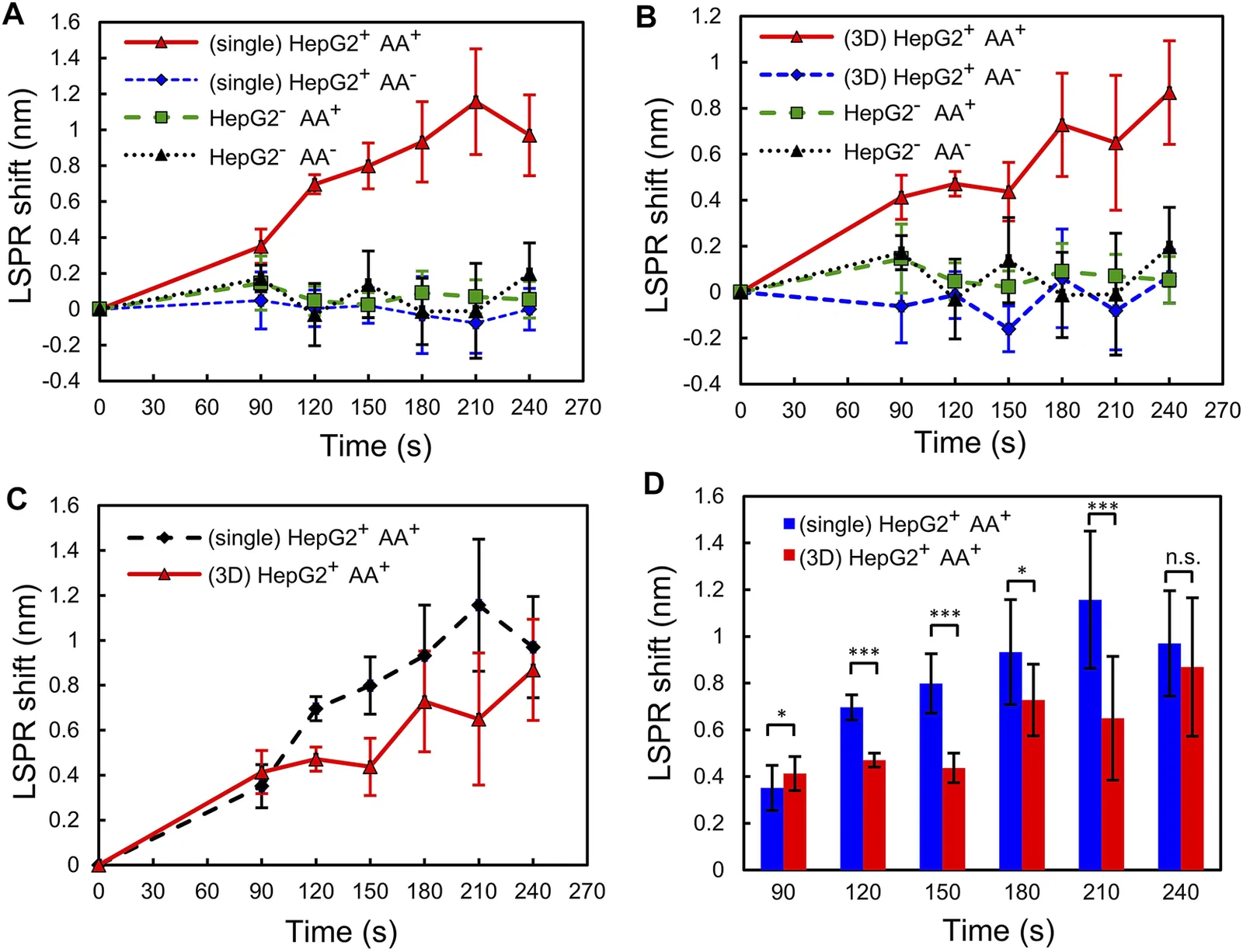
Detection of H_2_O_2_ released from the single cells and the 3D spheroids. LSPR shifts of the plasmonic gel films incubated with sample solutions taken from **(A)** the single cells and **(B)** the 3D spheroids at different time points. The four groups of the experiment: (HepG2^+^ AA^+^) HepG2 cells stimulated by 1.5 μm AA; (HepG2^+^ AA^−^) HepG2 cells not stimulated by 1.5 μm AA; (HepG2^−^ AA^+^) cell-free MEM culture medium mixed with 1.5 μm AA; (HepG2^−^ AA^−^) cell-free MEM culture medium only. **(C,D)** Comparison of the responses to the AA stimulation between the single cells and the 3D spheroids. Error bars show standard deviations from 3 cell experiments, each of which was measured 5 times using the gel film. n.s: not significant, *p < 0.05, **p < 0.01, ***p < 0.001.

Finally, to investigate the difference in the responses to the AA stimulation between HepG2 single cells and 3D spheroids, we use 1.5 μm AA to stimulate both of them and then use the plasmonic gel film to perform time-lapse detection of H_2_O_2_ in the complete culture media. The same detection is performed for the cells cultured in both culture conditions without AA stimulation for comparison. In addition, cell-free complete culture media with and without 1.5 μm AA are measured as the control groups. For the time-lapse monitoring of H_2_O_2_ secretion, we take a sample of the cell culture medium every 30 s after the AA stimulation and then perform measurement with the plasmonic gel film. Culture media of the control groups and the cells that are not stimulated by AA are sampled and measured at the same time during the time-lapse measurement for comparison. The results, as shown in Figures 5A,B, show that the concentrations of H_2_O_2_ in the culture medium of both the single cells and the 3D spheroids increase with time after the AA stimulation. The maximum LSPR shifts measured within 240 s after AA stimulation are ∼1.2 nm and ∼0.9 nm for the single cells and the 3D spheroids, respectively. According to the calibration curve shown in Figure 4B, the LSPR shifts of 1.2 nm and 0.9 nm corresponds to ∼1–2 μm H_2_O_2_. In contrast, the HepG2 cells that are not stimulated by AA and the cell-free control groups, either with or without AA, do not secrete a measurable amount of H_2_O_2_ during the same period. The coefficient of variation (standard deviation/mean) of the calibration measurement at similar wavelength shift is about 15.4%. Therefore, the errors of the measured H_2_O_2_ concentrations in the experiments are estimated to be around 15.4%. In addition, the results also suggest that the refractive index variation of the medium during the cell culture was minimal and has minimal effects on the LSPR wavelength shift. Figures 5C,D show the comparison of the responses to the AA stimulation between the single cells and 3D spheroids. The unpaired Student’s t-test is performed to compare the results obtained from the experiments in which the cells are cultured in different formats. The results show that the single cells secret more H_2_O_2_ than the 3D spheroids after the AA stimulation.

## Discussion

The experimental results confirm the feasibility of the time-lapse H_2_O_2_ measurement from the complete medium collected from the cell culture. In addition, the quantitative measurement results show the different temporal H_2_O_2_ secretion between single cell and spheroid culture formats. During the AA stimulation, the H_2_O_2_ concentration in single-culture culture increases in a faster pace and reaches to a higher value comparing to that in spheroid culture. The H_2_O_2_ concentrations in both culture formats become similar after 4 min stimulation. The observation suggests that the culture format does affect the H_2_O_2_ secretion kinetics and maximum concentration; however, the format has less effects on the H_2_O_2_ concentration after an extended period. The slower H_2_O_2_ concentration increase may be resulted from the diffusion of AA through the 3D cell arrangements. In addition, it has been reported that oxygen tension can be greatly reduced within a spheroid due to limited diffusion and cell metabolism (Sarkar et al., 2018; Close and Johnston, 2021). The low oxygen tension may also impede the H_2_O_2_ production by the cells (Maddalena et al., 2017). The simultaneous measurement of oxygen tension and H_2_O_2_ can better help to elucidate the underlying molecular mechanisms.

Furthermore, AA has been intensively investigated as an anti-cancer drug and its roles in cancer treatment in recent decades. It has been identified that pharmacologic AA concentrations are capable of selectively killing cancer cells though various mechanisms. Among them, two mechanisms of anti-cancer activity with AA are well recognized: H_2_O_2_-induced oxidative stress and DNA demethylation mediated by activation (Shenoy et al., 2018). AA has been found to be able to generate and deliver H_2_O_2_ to tissues and in extracellular fluid based on *in vitro* cell culture and *in vivo* studies, and to decrease growth of aggressive tumor in animal experiments (Chen et al., 2005; Chen et al., 2007; Chen et al., 2008; Gao et al., 2019). However, due to the technical limitations, the *in vitro* experiments are limited to in-direct or end-point H_2_O_2_ measurement without kinetic information. Furthermore, comparison of the H_2_O_2_ generation between different cell culture formats are not systematically investigated. The integrated approach demonstrated in this paper opens a new window to better studied the H_2_O_2_ generation and variation in complete cell culture medium in a straightforward and rapid manner, which can greatly help scientists to investigate H_2_O_2_ related biomedical phenomena.

## Conclusion

In this paper, an integrated approach is developed to compare the H_2_O_2_ secretion from living hepatocytes cultured in single cells and spheroids. The uniformed-sized cell spheroids are formed and cultured in the developed microfluidic device. The device allows the cell spheroids cultured in a reliable manner with easy medium exchange, and the spheroids can be harvested from the device with great integrity for the following analysis. The H_2_O_2_ in the complete cell culture medium is measured using a hydrogel-based LSPR substrate. Taking advantage of the enzyme-catalyzed chromogenic reaction and hydrogel-based substrate, the H_2_O_2_ can be detected with high specificity in a straightforward manner for the time-lapse measurement. The H_2_O_2_ secreted from the living cells (HepG2) cultured in single cells and spheroids are compared, and the results show the different secretion temporal patterns confirming the important roles of the culture formats. The developed approach provides a simple and useful tool to study H_2_O_2_ related biomedical topics.

## Data Availability

The raw data supporting the conclusion of this article will be made available by the authors, without undue reservation.
